# Supplementary figures and images for: Non-canonical functions of a mutant TSC2 protein in mitotic division
Source: PLoS One. 2023 Oct 4;18(10):e0292086. doi: 10.1371/journal.pone.0292086 (PMC10550124; doi:10.1371/journal.pone.0292086)

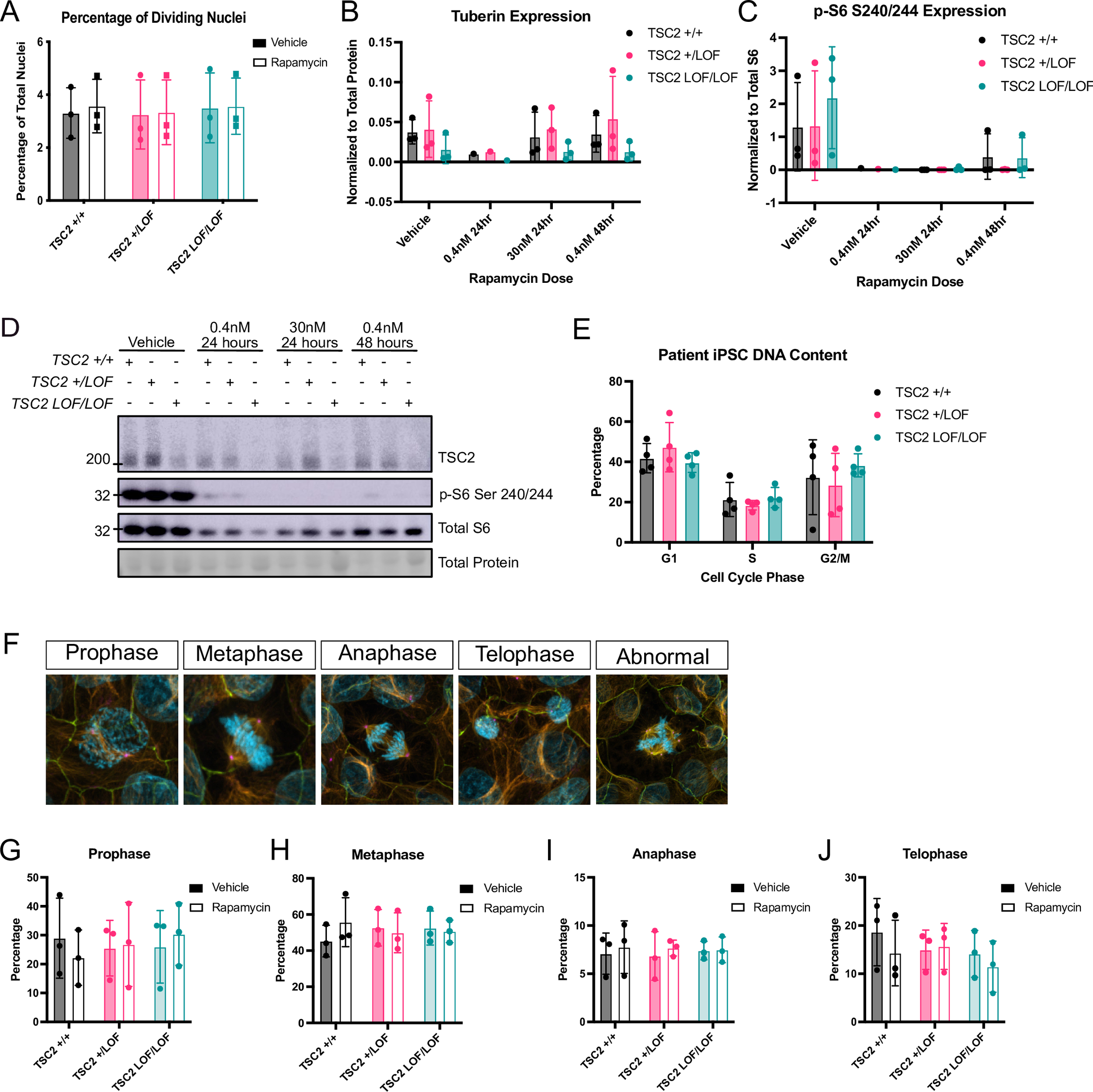

Supplement: S1 Fig — (a) Quantification showing average percentage of cells in mitosis per genotype and treatment for experiments included in Fig 1. N = 3 independent replicates per genotype and treatment, [ANOVA]. Error bars = SD. (b) Quantification of relative expression of tuberin in immunoblot shown in d. N = 3 independent replicates, paired students t test. Error bars = SD. (c) Quantification of relative expression of p-S6 Ser240/244 in immunoblot shown in d. N = 3 independent replicates, paired students t test. Error bars = SD. (d) Representative immunoblot showing protein expression of tuberin and p-S6 in patient iPSCs. (e) Quantification showing average percentage of cells in cell cycle phase as determined by DNA content per genotype. N = 3 independent replicates per genotype, [ANOVA]. Error bars = SD. (f) Example images of patient iPSCs showing expression of TUBA (yellow) and Hoechst (teal) to identify DNA to display each cycle of mitosis. (g-j) Quantification showing average percentage of cells in each mitotic phase as determined by mitotic indexing per genotype and treatment. N = 3 independent replicates per genotype, [ANOVA]. Error bars = SD. (TIF) [file pone.0292086.s001.tif]

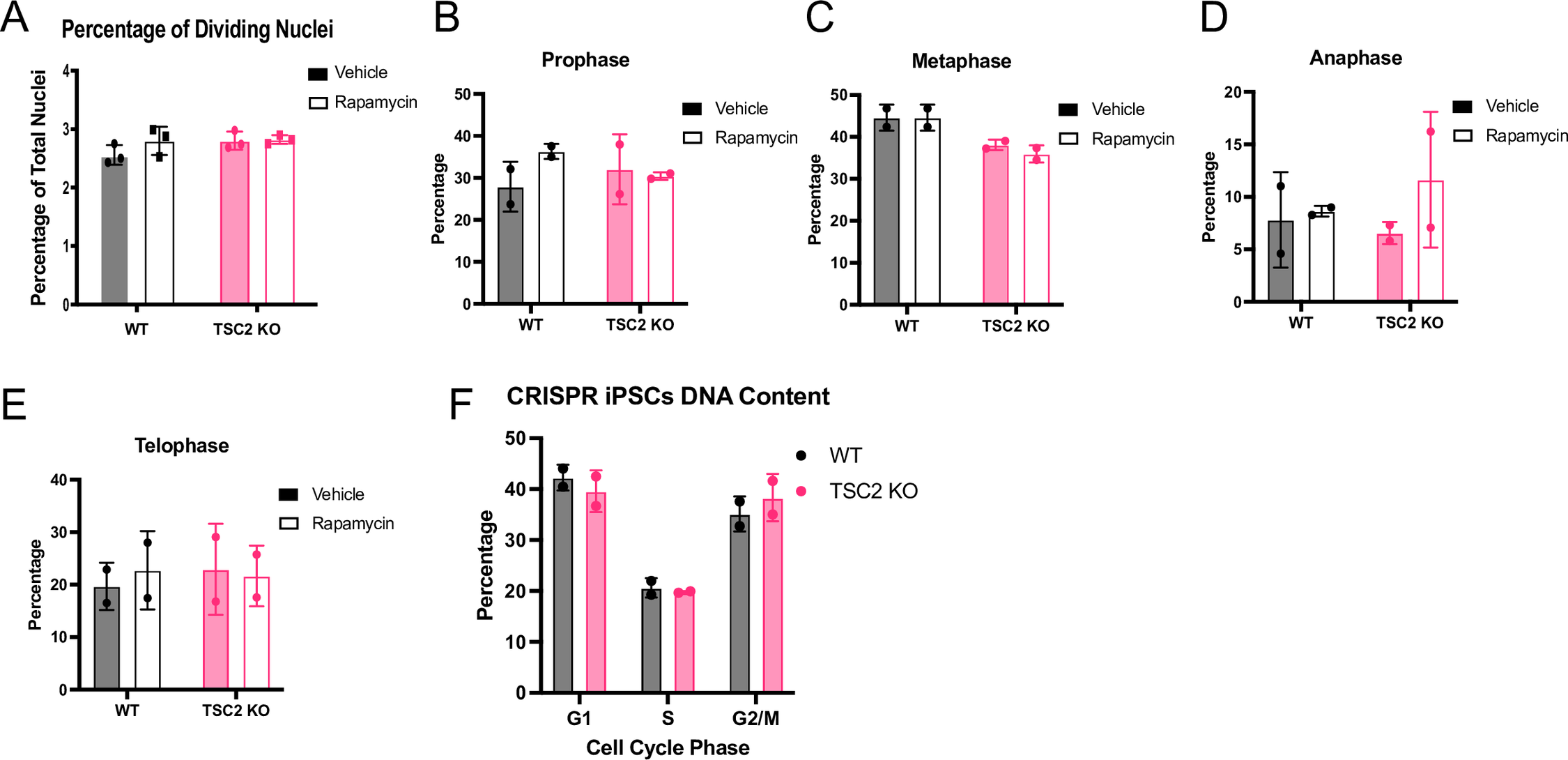

Supplement: S2 Fig — (a) Quantification showing average percentage of cells in mitosis per genotype and treatment for experiments included in Fig 1. N = 3 independent replicates per genotype and treatment, [paired students t test]. Error bars = SD. (b-e) Quantification showing average percentage of cells in each mitotic phase as determined by mitotic indexing per genotype and treatment. N = 2 independent replicates per genotype. Error bars = SD. (f) Quantification showing average percentage of cells in cell cycle phase as determined by DNA content per genotype. N = 2 independent replicates per genotype. Error bars = SD. (TIF) [file pone.0292086.s002.tif]

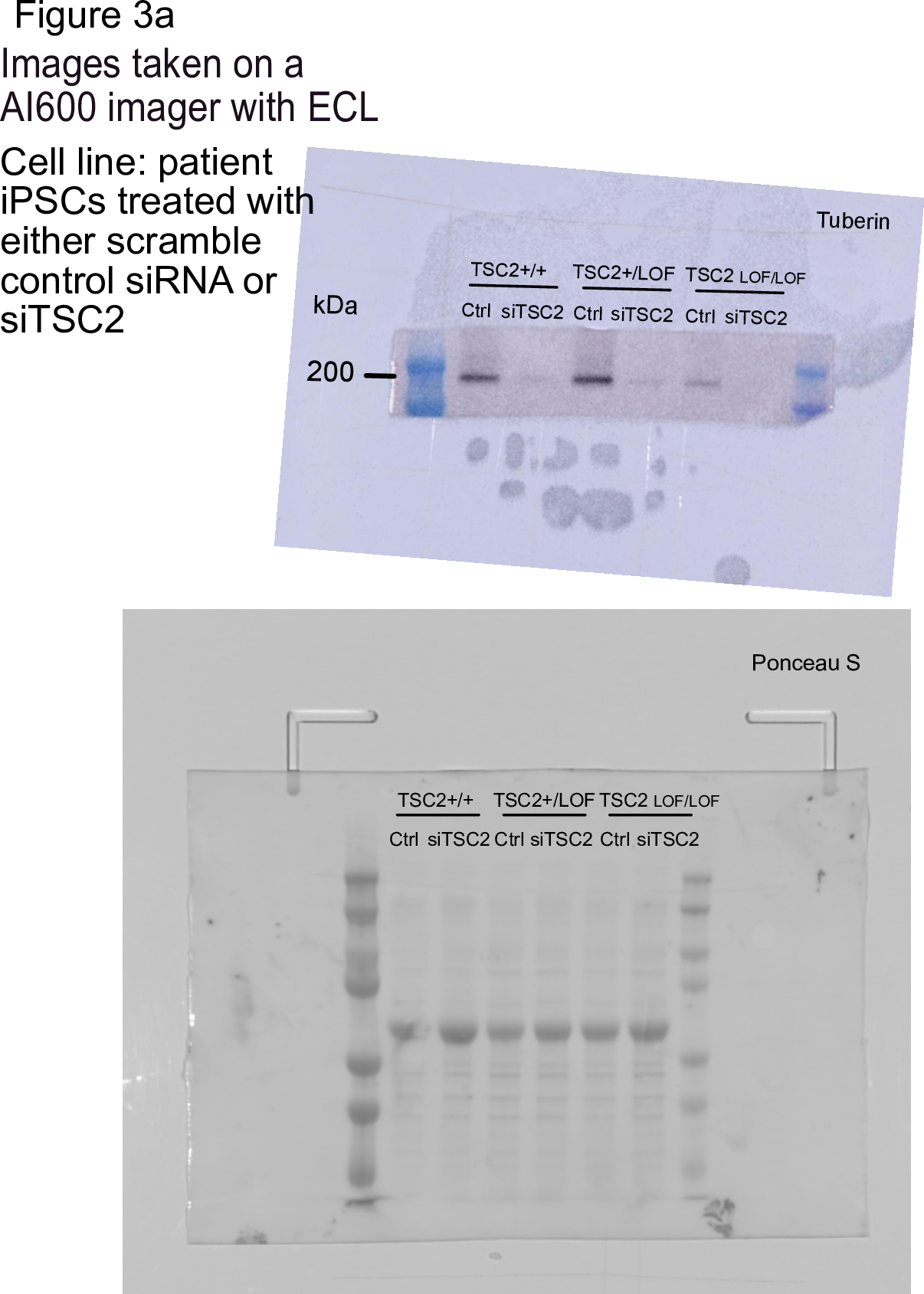

Supplement: S1 Raw images — (ZIP) [file pone.0292086.s003.zip › S1_raw_iamges (3).tif]

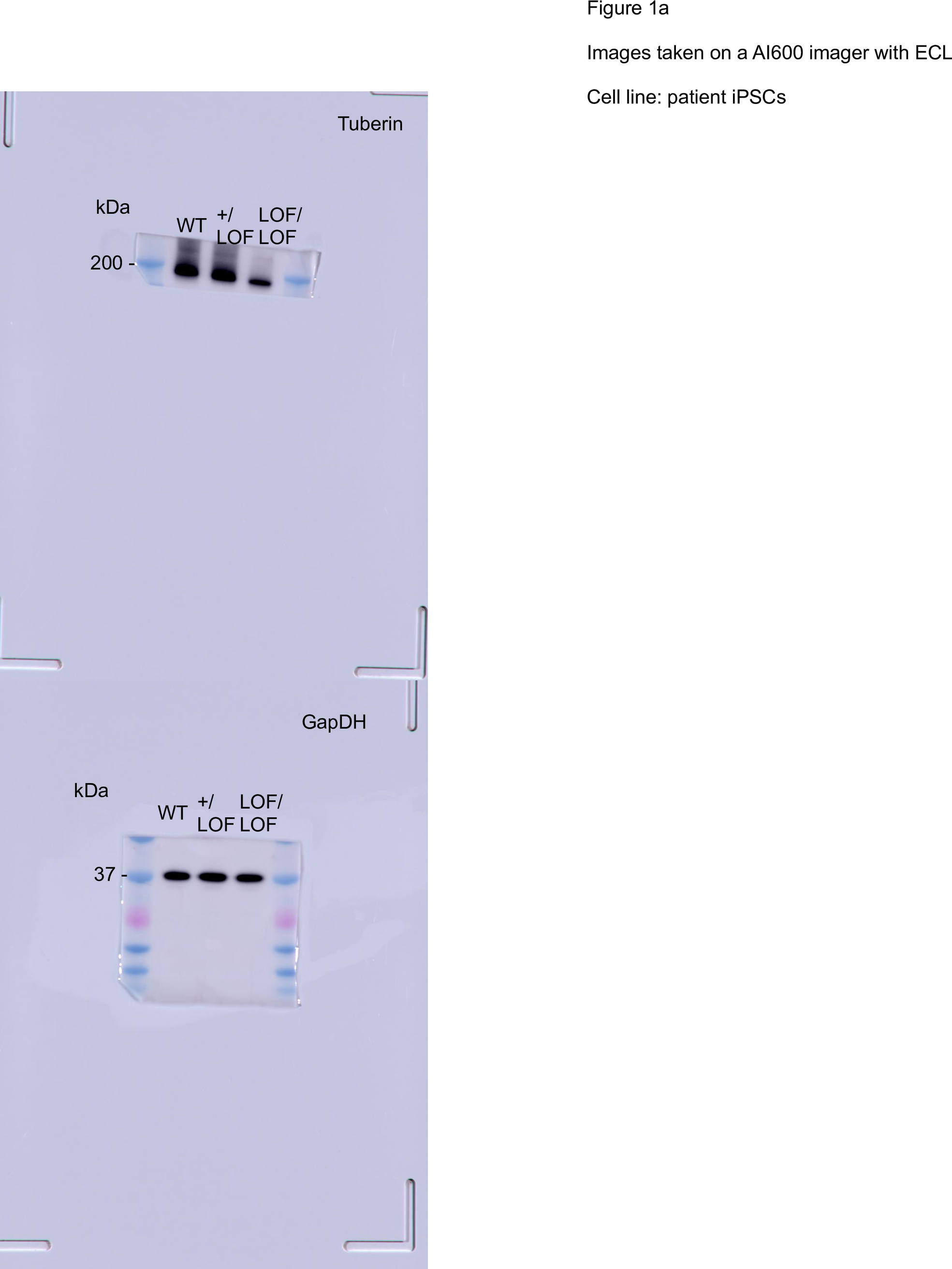

Supplement: S1 Raw images — (ZIP) [file pone.0292086.s003.zip › S1_raw_iamges (1).tif]

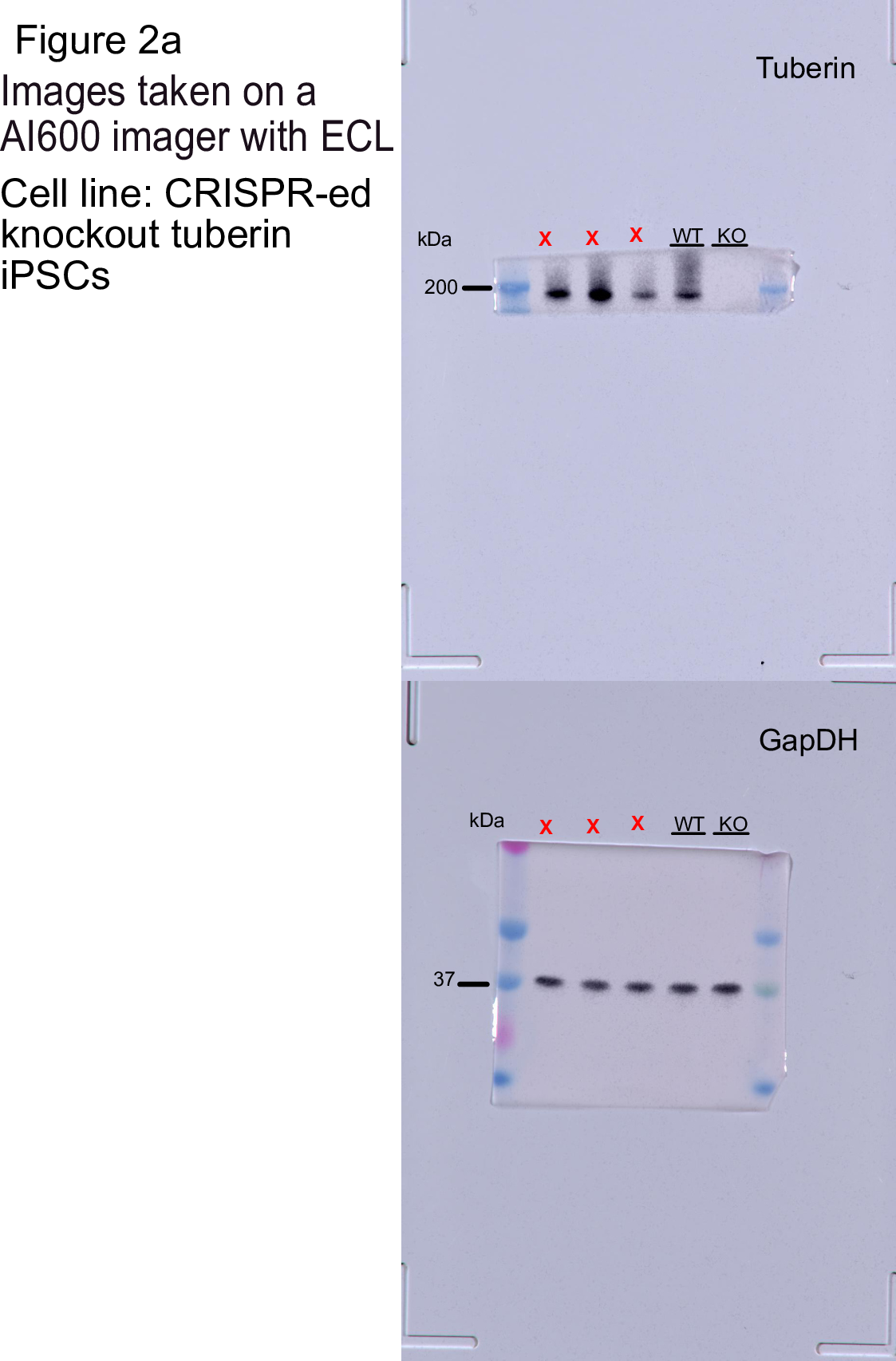

Supplement: S1 Raw images — (ZIP) [file pone.0292086.s003.zip › S1_raw_iamges (2).tif]
